# The association between vancomycin trough concentrations and acute kidney injury in the neonatal intensive care unit

**DOI:** 10.1186/s12887-017-0777-0

**Published:** 2017-02-11

**Authors:** Vidit Bhargava, Michael Malloy, Rafael Fonseca

**Affiliations:** 1 Department of Pediatrics, University of Texas Medical Branch, 301 UNIVERSITY BLVD, GALVESTON, 77555 TEXAS USA; 2 Department of Pediatrics, Division of Neonatology, University of Texas Medical Branch, 301 UNIVERSITY BLVD, GALVESTON, 77555 TEXAS USA

**Keywords:** AKI, Nephrotoxicity, Prematurity, Vancomycin

## Abstract

**Background:**

Vancomycin has recently gained popularity as an empiric therapy for late onset sepsis in the NICU. Changes in resistance patterns in common organisms has resulted in targeting higher trough concentrations of vancomycin. Consequently, an increase in vancomycin associated nephrotoxicity has been speculated. The objective of this study is to compare the incidence of acute kidney injury (AKI) in neonates with serum vancomycin trough concentrations less than 10 mg/L, 10–15 mg/L, or greater than 15 mg/L.

**Methods:**

A retrospective chart review of patients in the neonatal intensive care unit (NICU) was conducted to determine the incidence of AKI in neonates receiving vancomycin.

**Results:**

The overall incidence of AKI was 2.7%. Comparison of the incidence of AKI in the three groups using Mantel-Haenszel Chi-Square test showed a statistically significant association between increasing vancomycin trough concentration and incidence of AKI.

**Conclusion:**

There is a low incidence of AKI in neonates receiving vancomycin. However, there is a positive correlation between increasing vancomycin trough concentrations and an increasing serum creatinine.

**Electronic supplementary material:**

The online version of this article (doi:10.1186/s12887-017-0777-0) contains supplementary material, which is available to authorized users.

## Background

Vancomycin is a glycopeptide antibiotic which gained popularity in 1980’s for treatment of coagulase negative Staphylococcus (CONS) and Methicillin resistant staphylococcus aureus (MRSA). Late onset sepsis is a common concern in premature infants in neonatal intensive care unit (NICU). Vancomycin is widely used as an empiric therapy for late onset sepsis, and in confirmed infections with CONS and MRSA. [1, 2] Consequently, vancomycin is used in the NICU even though limited information is available concerning the dosing, monitoring, and adverse effects of this medication in neonates. Additionally, increasing antibiotic resistance among familiar pathogens in the NICU, as evidenced by higher minimum inhibitory concentrations (MICs), has resulted in targeting higher vancomycin trough concentrations. [3, 4] Vancomycin associated nephrotoxicity has not been well studied in the neonatal population and limited data exists on the association between higher vancomycin trough and incidence of acute kidney injury (AKI).

This study aims to measure the association between increasing trough concentrations and AKI in neonates receiving vancomycin therapy. We also look at the effect of co-administration of other nephrotoxic agents on the incidence of AKI.

## Methods

A retrospective chart review was performed for patients in NICU at University of Texas Medical Branch (UTMB) at Galveston. The electronic medical record was reviewed between January 2008 and December 2012 to determine the incidence of AKI in neonates receiving vancomycin. The patient population consisted of premature neonates admitted to NICU at UTMB and received at least one course of vancomycin. Each patient may have received several courses of vancomycin during this period. Courses of vancomycin were included in the study group if they met the following inclusion criteria: a) Duration of treatment at least 5 days b) Availability of serum creatinine (SCr) values both prior to and after completing the vancomycin therapy and c) at least one vancomycin trough collected during the duration of treatment. Courses of vancomycin therapy were excluded if: a) evidence of pre-existing renal insufficiency or congenital anomalies including renal agenesis, renal hypoplasia, polycystic kidney disease, or renal dysplasia were present b) extracorporeal membrane oxygenation was required or c) there was incomplete data in the UTMB electronic medical record, Epic. Baseline serum creatinine was defined as the serum creatinine obtained prior to starting vancomycin therapy. Post vancomycin creatinine was defined as the serum creatinine value obtained at the end of vancomycin therapy or after a suspected episode of AKI.

Courses of vancomycin therapy were divided into three groups based on highest achieved vancomycin trough concentrations; less than 10 mg/L, 10–15 mg/L, or greater than 15 mg/L. Standardized vancomycin dosage proposed by Capparelli et al., based on gestational age was used for each patient.[5] The trough was obtained prior to the fourth dose. If the trough was found sub-therapeutic or toxic, changes in dose or frequency of vancomycin administration were accordingly made. The change in dose was subsequently followed by a repeat trough measurement prior to the 4^th^ dose. For each course of vancomycin therapy, multiple vancomycin trough concentrations may have been measured, but the highest achieved trough concentration was used to classify courses of vancomycin therapy into the three study groups. The incidence of AKI was determined in each group. AKI was defined as an increase in SCr of at least 0.5 mg/L or an increase of at least 100% from lowest trough previously available. This definition is based on pRIFLE criteria for renal injury proposed by Akcan-Arikan et al. and the increase in serum creatinine proposed by moghal et al. [6–8] A decrease in urine output was not used as a defining criteria due to lack of this being a universal finding.

Data collected included gestational age; postnatal age; gender; birth weight; APGAR scores; vancomycin dose, frequency, duration, and trough concentrations; date and time of vancomycin doses and trough concentrations; concurrent nephrotoxic medications administered including amphotericin B, acyclovir, amikacin, captopril, dobutamine, dopamine, enalapril, epinephrine, ganciclovir, gentamicin, indomethacin, ibuprofen, naficillin, and tobramycin; blood culture results; type of infection; presence of a patent ductus arteriosus; and final discharge status. Maternal history was also collected and included the presence of pregnancy-induced hypertension, chorioamnionitis, diabetes, renal dysfunction, or urinary tract infection.

Mantel-Haenszel Chi-Square test was used to compare the incidence of AKI between the three groups. A *p*-value of < 0.05 was considered to be statistically significant. Regression analysis was used to examine the relationship between vancomycin trough concentrations and serum creatinine. All statistical analyses were done using SAS 9.3©. The study was approved by the institutional review board (IRB) at UTMB, Galveston.

## Results

Nine hundred and sixty-two patients receiving vancomycin therapy administered between January 2008 and December 2012 were evaluated for study inclusion. Eight hundred and fifty-two patients were excluded due to inability to meet one or more inclusion criteria. The majority of patients were excluded due to vancomycin being administered for less than five days. The second most common reason for exclusion was incomplete data in the electronic medical record. Therefore, 110 patients were included in the analysis. The majority of patients were male. The mean birth weight was 1200 g, and the mean gestational age was 29 weeks. (Table 1) Central line associated blood stream infection (CLABSI), sepsis and necrotizing enterocolitis (NEC) were the most common suspected diagnoses for which patients were started on vancomycin therapy. The most common organism was Coagulase Negative Staphylococcus aureus (32 patients) followed by Enterococcus faecalis (5 patients) and Staphylococcus aureus.

**Table 1:** Baseline characteristics of patients

*N* = 110	
Male/Female	58/52
Birth weight (grams) ± SD	1200 ± 734
Gestational age (weeks) ± SD	29 ± 5
Postnatal age at time of first vancomycin course (days) ± SD	23 ± 27

**Fig. 1:**
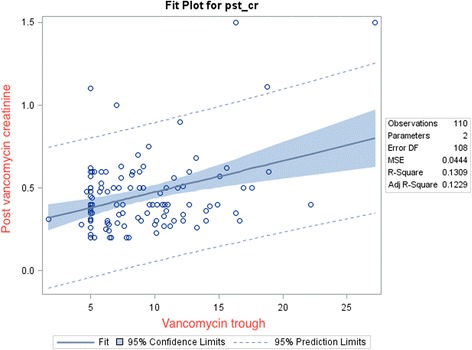
Showing a Fit plot between vancomycin trough concentrations and post vancomycin creatinine. The plot depicts a positive co-relation between the two parameters

One hundred ten patients were further studied to determine an association with AKI. There were 72 patients with a highest vancomycin trough concentration less than 10 mg/L, 27 patients with a highest vancomycin trough concentration of 10–15 mg/L, and 11 patients with a highest vancomycin trough concentration greater than 15 mg/L. The incidence of AKI was 1.39% (1/72 patients) in the group achieving vancomycin trough concentrations less than 10 mg/L, 0% (0/27 patients) in the group achieving vancomycin trough concentrations between 10–15 mg/L, and 18.18% (2/11 patients) in the group achieving vancomycin trough concentrations greater than 15 mg/L (Table 2). There was a statistically significant association between AKI and vancomycin trough groups (*p* = 0.04). Regression analysis between increasing vancomycin trough concentrations and post vancomycin serum creatinine values demonstrated a positive correlation value of 0.32 (*p* < 0.05) (Fig.1).

**Table 2:** Incidence of acute kidney injury

Group	Total number of patients	Patients with AKI	Incidence of AKI
Vancomycin trough < 10 mg/L	72	1	1.38%
Vancomycin trough 10–15 mg/L	27	0	0%
Vancomycin trough > 15 mg/L	11	2	18.18%
TOTAL	110	3	2.7%

Each of the patients with AKI were also receiving at least one concurrent nephrotoxic medication including dobutamine, dopamine, and/or gentamicin (Table 3). Gentamicin was the only nephrotoxic drug that a large percentage of the 87 patients received during vancomycin therapy (79%). There was no significant correlation between post vancomycin creatinine values and the total number of days’ gentamicin was received (*p* = 0.10) or of a change in creatinine of greater than or equal to 0.5 mg/dl from pre- to post-vancomycin administration (*p* = 0.13). In an ANOVA regression equation with gentamicin days run as a covariate with vancomycin trough group, however, gentamicin days were significantly associated with post vancomycin creatinine values (*p* = 0.04). The vancomycin trough groups remained significantly associated with post vancomycin creatinine values independent of gentamicin days (*p* < 0.01).

**Table 3:** Cases of acute kidney injury

Case	Trough (mg/L)	Timing of trough	Dose of vancomycin	Frequency	Duration of treatment	Total vancomycin days
1	<5.0	Prior to 4^th^ dose	20 mg/kg 20 mg/kg	Q18H Q12H	4 days 5 days	9 days
2	18.8	Prior to 4^th^ dose	20 mg/kg	Q24H	6 days	6 days
3	27.2	Prior to 4^th^ dose	20 mg/kg 15 mg/kg	Q24H Q24H	5 days 6 days	11 days

## Discussion

There has been an on-going debate on the dosage, frequency of administration and the most appropriate monitoring of vancomycin therapy. This is the result of multiple factors responsible for clearance of vancomycin including gestational age, post-natal age, weight, renal tubular function and creatinine levels. [9] Historically, area-under-the-curve concentration versus time to the minimum inhibitory concentration (AUC:MIC), was accepted as the pharmacokinetic and pharmacodynamic predictor of adequate treatment with vancomycin. AUC: MIC > 400 has been recommended to achieve desired anti-microbial effect in both adult and pediatric populations. [10] A consensus statement released by the American Society of Health-System Pharmacists, the Infectious Diseases Society of America, and the Society of Infectious Diseases Pharmacists in 2009 recommended vancomycin as the first choice drug for MRSA with MIC < 2. [10] The consensus statement also recommends assessment of vancomycin clinical effectiveness through serum vancomycin trough concentrations at steady state. Studies have demonstrated that trough between 7–10 mg/L corresponds to AUC:MIC > 400 if the MIC of MRSA is < 1. [11] However, with increasing MIC’s higher trough concentrations are recommended. In serious clinical infections such as endocarditis, meningitis, hospital-acquired pneumonia, bacteremia, and osteomyelitis trough concentrations between 15–20 are recommended.[12] These higher troughs allow for greater vancomycin exposure, a higher AUC and the ability to reach the goal AUC:MIC ≥ 400, or a trough concentration approximately four to five times the MIC of the infecting organism. [12] Concerns for increased nephrotoxicity, an adverse effect traditionally associated with vancomycin, has accompanied the recommendation for more aggressive dosing.

In adults, increasing serum vancomycin trough concentrations have been associated with increasing incidence of AKI. In a study done to evaluate vancomycin-associated nephrotoxicity incidence in adults with MRSA infections with serum trough concentrations of 15–20 mg/L and receiving concomitant nephrotoxic medications, a significantly higher incidence of AKI was noted.[3] A 2011 prospective study also showed similar results. Adults with MRSA infections had a greater risk of AKI with serum vancomycin trough concentration greater than 15 mg/L.[13] Similarly, adults with MRSA pneumonia were shown to be at a 3–5 times greater risk for developing AKI with vancomycin serum trough concentrations of 15 mg/L.[14] On the other hand, there are other studies in adults which did not show increased risk of AKI with elevated serum vancomycin trough > 15 mg/L. In study done by Prabaker et al., an overall incidence of 2.1% was noticed with vancomycin serum trough concentration between 15–20 mg/L [15].

Nephrotoxicity in the neonatal population is poorly defined. Several definitions of acute kidney injury (AKI) exist in the literature; however, there are no standard criteria for diagnosing AKI in neonates. Commonly used definitions of AKI in neonates include oliguria of less than 1 mL/kg/h of urine output that develops 24 h after birth and persists for at least 24 h, an increase in serum creatinine (SCr) to greater than 1.5 mg/L 72 h after birth, or an increase in SCr between 0.5 and 1 mg/L per day.[8, 16] Nephrotoxicity attributed to vancomycin use is not clearly defined in neonates; however, the mechanism of injury to the kidney is believed to be the result of proximal tubule damage. [17, 18] Although causality has not been proven, a higher risk of vancomycin-induced nephrotoxicity in the pediatric population with higher vancomycin trough concentrations has been speculated.

Due to pharmacokinetic differences between adults and neonates, and since neonates have immature renal function compared to adults, the applicability and adverse effects of increased target troughs in premature neonates remain unknown. Lastly, it has been documented in adult populations that concurrent treatment using other known nephrotoxic agents, such as aminoglycosides, is associated with an increased risk for nephrotoxicity with vancomycin. [19] Again, it is unclear whether this is true in the neonatal population.

Previously done studies in children receiving vancomycin alone have reported incidence of AKI from 9%–14%. Linder et al. and Nahata et al. found the incidence of AKI in neonatal population to be low. Their studies did not show statistically significant difference in the incidence of AKI with concomitant administration of gentamicin.[20, 21] However, McKamy and Knoderer et al. reported slightly higher overall incidence of AKI at 14% in children between the ages 1 month and 17 years.[17, 22] Both the studies also reported significantly higher incidence of AKI with vancomycin trough > 15 mg/L at 28% and 18% respectively.

In our study, the overall incidence of AKI was low at 2.71%. A statistically significant association between AKI and vancomycin trough groups was found (*p* = 0.04). There was also a positive correlation between vancomycin trough concentrations and post vancomycin creatinine values, indicating that vancomycin may have some role in AKI in predisposed individuals. It also indicates that higher vancomycin troughs are associated with rising creatinine values post treatment. Additionally, we looked at several covariates using a linear regression model to assess whether these were significantly associated with higher post vancomycin creatinine values. Highest vancomycin trough value (*p* < 0.001), total vancomycin days (*p* ~ 0.0021) and gestational age (*p* value < 0.001) were found to have an independent association. Gender and APGAR scores were also looked at and were not found to be significantly associated with vancomycin trough and rising creatinine values. Even after controlling for these independent variables, the highest trough value and post vancomycin creatinine values showed a significant association.

Also of interest is the observation that vancomycin trough groups continued to be positively associated with increasing post vancomycin creatinine values independent of the number of days of gentamicin the infants had received. In addition, independent of vancomycin trough groups there was a positive association between the number of days that gentamicin was received and post vancomycin creatinine values. Thus, gentamicin, another potentially nephrotoxic had its effect on post vancomycin creatinine values obscured by the effect of vancomycin. Only after having the effect of vancomycin controlled for in the regression equation was gentamicin’s effect revealed. Because of the small number of courses associated with our definition of acute kidney failure whether or not vancomycin and gentamicin were additive in their nephrotoxic effects cannot be determined in this analysis. Larger studies are needed to confirm these findings.

Our study has limitations. The study was retrospective in nature and had a small sample size. Due to the lack of a standardized definition a hybrid definition of AKI was used in this study. Another limitation is that patients may have received multiple courses of vancomycin therapy during the same admission. However, courses of vancomycin therapy were often separated by weeks. Further, for each course of vancomycin therapy, multiple vancomycin trough concentrations may have been measured, but the highest achieved trough concentration was used to divide the vancomycin courses into the three study groups. Finally, despite the fact that patients were often on concurrent nephrotoxic medications, only three cases of AKI were identified in this study which is different from the previously reported studies.

## Conclusion

Based on our study, vancomycin troughs greater than 20 mg/dl may be associated with increased incidence of AKI. We recommend close monitoring of vancomycin trough concentrations during therapy and appropriate alteration in dosage/frequency should be made based on serum trough concentrations. However, this was a single-center retrospective study, and larger prospective studies are required to validate this finding.

## Additional files

Additional file 1: The file contains raw data used for analysis during the study. This includes patient gender, prenatal history, birth history, vancomycin start and end dates for each course of vancomycin administered. It also contains information regarding vancomycin trough and serum creatinine values obtained during each vancomycin course. (XLSX 55 kb)

## Abbreviations

AKI: Acute kidney injury
AUC: MIC: Area-under-the-curve concentration versus time to the minimum inhibitory concentration
CONS: Coagulase negative staphylococcus aureus
MRSA: Methicillin resistant staphylococcus aureus
NICU: Neonatal intensive care unit
SCr: Serum creatinine
UTMB: University of Texas Medical Branch
